# Quantum paradigm of the foldover magnetic resonance

**DOI:** 10.1038/s41598-021-87196-w

**Published:** 2021-04-07

**Authors:** Yu. M. Bunkov, A. N. Kuzmichev, T. R. Safin, P. M. Vetoshko, V. I. Belotelov, M. S. Tagirov

**Affiliations:** 1 M-Granat, Russian Quantum Center, Bolshoy Bulvar, 42, Skolkovo, Moscow, Russia 121205; 2 Kazan Federal University, Kazan, Russia 420008; 3 Lomonosov Moscow State University, Leninskie gori, Moscow, Russia 119992

**Keywords:** Quantum physics, Bose-Einstein condensates

## Abstract

The explosive development of quantum magnonics requires the consideration of several previously known effects from a new angle. In particular, taking into account the quantum behavior of magnons is essential at high excitations of the magnetic system, under the conditions of the so-called phenomenon of “foldover” (bi-stable) magnetic resonance. Previously, this effect was considered in the quasi-classical macrospin approximation. However, at large angles of magnetization precession, the magnon density exceeds the critical value for the formation of a magnon Bose condensate (mBEC). Naturally, this purely quantum phenomenon does not exist in the classical approximation. In addition, mBEC leads to superfluid transfer of magnetization, which suppresses the macroinhomogeneity of the samples. The experiments presented in the article show that quantum phenomena well describes the experimental results of nonlinear magnetic resonance in yttrium iron garnet. Thus, we remove the questions that arose earlier when considering this effect without taking into account quantum phenomena. This discovery paves the way for many quantum applications of supermagnonics, such as the magnetic Josephson effect, long-range spin transport, Q-bits, quantum logic, magnetic sensors, and others.

## Introduction

The aim of our research is experimental investigations of nonlinear magnetic resonance under conditions of strong excitation and a modern analysis of this phenomenon, taking into account the results of breakthrough discoveries of coherent transport of magnons by spin supercurrent at their high concentration. Usually, the dynamic properties of the deflected magnetization are described in terms of the Landau–Lifshitz–Gilbert phenomenological model. This approach uses the laws of classical mechanics and correctly describes magnetic systems at low levels of excitation. According to the Holstein–Primakoff transformation^1^, a magnetically ordered system consists of a ground state (which plays the role of quantum vacuum) and a gas of quantum excitations–magnons, which obey Bose statistics. The density of magnons can change due to their creation and annihilation from the quantum vacuum. At a finite temperature, the concentration of thermally activated magnons is low, and the spin dynamics can be described in the classical approximation. However, the concentration of magnons can be significantly increased by excitation of non-equilibrium magnons by methods of magnetic resonance. According to Bose statistics, magnons should form a Bose–Einstein condensate (mBEC) at a high concentration and obey the laws of quantum physics. In this case, quantum transport phenomena such as superfluidity must be considered.

The first mBEC state was experimentally discovered in the antiferromagnetic superfluid $$ ^3$$He-B as a state emitting a very long-lived induction signal even in a strongly inhomogeneous magnetic field^2,3^. In these experiments, exciting radio frequency (RF) pulses produced a high density of magnons, which radiate from the beginning, a typical induction decay signals. They decay is due to the inhomogeneity of the effective magnetic field. However, with a certain time delay, the coherence of magnons spontaneously recovers, and they emit very long coherent signals. It can last for a few minutes if mBEC is protected topologically^4,5^. These signals are now considered as a time crystals^6^.

It was found that mBEC can be maintained permanently when the relaxation (evaporation) of magnons is compensated by the creation of new magnons from a quantum vacuum using high frequency pumping^7,8^. According to Feynman, new magnons are emitted with the wave function of the existing mBEC state^9^. In the case of repulsive interaction between non-equilibrium magnons, the mBEC state can exhibit the properties of quantum spin transport–spin superfluidity. The positive interaction energy is required to compensate for the kinetic energy of the magnon superflow. The balance between these energies determines the critical Landau flow of magnons and the critical gradient of phase $\nabla \alpha _c$, as well as the Ginzburg–Landau coherence length $\xi _{GL}$:

1 $$\begin{aligned} \nabla \alpha _c=1/\xi _{GL}=\sqrt{\omega _0 \Delta \omega } /c_{SW}, \end{aligned}$$

where $c_{SW}$ is a spin wave velocity and $\Delta \omega$ the frequency shift due to magnons interaction. The long distance spin supercurrent, Landau critical velocity, and phase slippages were observed in the experiments, described in^8,10^. Other superfluid phenomena have also been observed, such as the magnonic Josephson phenomena^11,12^ and magnon quantum vortices^13,14^. The quantum properties of magnonic BECs are described in a series of publications^15–17^. The comprehensive review of these phenomena can be found in^18,19^.

## Magnonic BEC

Observation of quantum spin phenomena in superfluid $$ ^3$$He-3 is quite expected since it is a quantum liquid. However, the question arose whether such quantum phenomena could be observed in solid magnetic materials. Essentially, magnetism is a quantum phenomenon because spin is of quantum origin. However, for many cases, magnetic phenomena can be described in the classical approximation, when a large ensemble of spins is described by phenomenological theories that take into account only the average value of the local magnetization. The phenomenon of spin transfer in solid magnets is sometimes considered by analogy with superfluid phenomena^20,21^. Despite the fact that its mathematical description has a formal similarity to superfluidity, it should be borne in mind that superfluidity and Bose condensation are purely quantum phenomena. Therefore, it makes no sense to consider them within the framework of classical models.

For description of magnetic quantum phenomena the paradigm of the Holstein-Primakov transformation should be applied^1^. Magnon quantum phenomena occur at a sufficiently high concentration of magnons at the conditions of Bose–Einstein condensat formation. Strictly speaking, the properties of mBECs are beyond the scope of classical physics and are traditionally described by the Gross-Pitaevskii formalism developed to describe the atomic Bose condensate^22^. The magnon density required for the formation of the Bose condensate is easy to calculate for various magnetically ordered substances, as shown in^23^.

A magnetically ordered state can be considered as a stationary ground state with a gas of thermal excitations–magnons, which are described by Bose statistics, since magnons have spin equal to 1. In this approach, magnetic resonance is a phased precession of the magnon cloud against the background of a stationary ground state. The non-excited magnons are in thermal equilibrium with the magnetic system. We can increase its density by pumping new, non-equilibrium magnons. In the “classical” consideration, this is described as a deflection of the magnetization $\mathbf {M}$ by an angle $\beta$. In the language of magnons, this process excites an additional magnons, the number of which can be estimated as:

2 $$\begin{aligned} \hat{\mathcal N}=\hat{a}^\dagger _0\hat{a}_0 = \frac{{\mathcal S}-\hat{\mathcal S}_z}{\hbar }~, \end{aligned}$$

where $\hat{a}^\dagger _0$ and $\hat{a}_0$ are operators of magnon annihilation and creation. This equation relates the number of excited magnons $\hat{\ N}$ to the deviation of spin ${\hat{S}}_z$ from its equilibrium value *S*.

As the magnon density increases, it can reach a critical value at which a Bose condensed state is formed^23^. This state can be described by the wave function:

3 $$\begin{aligned} \Psi = \sqrt{\frac{2S}{\hbar }} \sin \frac{\beta }{2} e^{i(\alpha + \omega t)} \,, \end{aligned}$$

where $\alpha , \, \, \beta$ and $\omega$ are the angle, phase and frequency of magnetization precession and deflection.

There are two approaches to study the thermodynamics of atomic systems: at fixed particle number *N* or at fixed chemical potential $\mu$. In the case of magnetic systems the role of the magnon chemical potential, $\mu_{\mathrm{M}}= dE/dN_{\mathrm{M}}$, where *E* is a energy of the system, is played by the frequency of precession $\omega =dE/dS_z$. This frequency is global—i.e. it is constant across the whole sample—in the same way as the chemical potential in the thermodynamic system. For the magnon BEC, these two approaches correspond to two different experimental arrangements: the pulsed and continuous one. In the case of free precession after the pulse, the number of magnons pumped into the system is conserved (if one neglects the losses of spin). This corresponds to the situation with the fixed $N_{\mathrm{M}}$, in which the system itself will choose the global frequency of the coherent precession—the magnon chemical potential, $\omega =\mu _{\mathrm{M}}$. The opposite case corresponds to a continuous wave resonance, when a small RF field is applied to compensate the relaxation. In this case the frequency of precession is fixed by the frequency of the RF field,

4 $$\begin{aligned} \mu _{\mathrm{M}}\equiv \omega = \omega _{\mathrm{RF}}, \end{aligned}$$

and now the number of magnons $N_{\mathrm{M}}$ will be adjusted to this frequency to match the resonance condition. This circumstance is especially important in the quantum description of foldover resonance, as will be shown below.

In 1989, a nonlinear bistable NMR signals were observed in experiments with $^3$He^8,24^. The properties of these signals are fundamentally different from those that should arise in the quasi-classical consideration of the spin system. Within the framework of quantum consideration, a magnon Bose condensate and a spatial redistribution of magnetization due to spin superfluidity arise at a sufficient density of nonequilibrium magnons. They lead to three main features of nonlinear signals.

1. In the gradient of magnetic field, spatial gradients of the precession phase arise, which lead to the appearance of superfluid currents. They redistribute the magnon density until the frequency shift compensates for the inhomogeneity of the magnetic field. As a result, the magnetization precesses spatially uniformly even in a strongly inhomogeneous magnetic field. This is a kind of analogue of Meissner effect, in which a superconducting current displaces the field from type I superconductors and makes the state of electron wave function uniform^25,26^.
2. The state of mBEC is determined by its chemical potential, which is given by the frequency of excitation, not by its amplitude. Accordingly, the relaxation rate of magnons and the energy absorbed from the radio frequency (RF) field are determined by the pump frequency and is independent on the RF field amplitude.
3. The mBEC state and the nonlinear magnetic resonance signal are destroyed when the amount of RF pumping is insufficient to maintain an appropriate magnon density. The relaxation rate of magnons usually depends on the magnon density, which increases with the displacement of the magnetic field from its resonance value. By measuring the field of disappearance of the signal at different pump powers, one can measure the dependence of the relaxation processes on the magnon density.

Naturally, the question arose about the applicability of the quantum mechanical consideration of the magnon condensate in the case of solid-state magnets. The magnetic properties of superfluid 3He are determined by its antiferromagnetic ordering and are not directly related to the properties of mass superfluidity. Within the framework of the Holstein–Primakov paradigm, a magnon gas is considered, which has all the properties of a Bose gas. In a number of works with solid-state magnets, such as CsMnF$_3$^27^ and MnCO$_3$^28^, similar quantum effects of magnonic superfluidity were observed. In these antiferromagnets, coupled nuclear-electron precession is observed. It was found that in the quasi-nuclear precession mode, the interaction of magnons leads to a strong frequency shift and the formation of a magnon Bose condensate. In addition to the properties listed above, non-resonant excitation of a Bose condensate was also found, when a sufficiently long RF pulse at a non-resonant frequency forms a Bose condensate of magnons with a chemical potential equal to the pumping frequency^29^. In addition, the formation of a Bose condensate of quasi-nuclear spin waves was directly demonstrated^30^. These results motivate a quantum mechanical consideration of the phenomenon of nonlinear resonance in YIG film, which also exhibits strong interactions between magnons leading to a dynamic frequency shift.

## Foldover magnetic resonance in YIG film

In this article, we set ourselves the task of showing that the properties of nonlinear magnetic resonance in YIG films are explained by the quantum properties of magnon BEC, which is formed when the magnetization deviates strongly from equilibrium. Early studies of magnetic resonance in YIG films discovered the effect of nonlinear resonance, sometimes referred to as bistable or foldover resonance. The essence of this resonance is that when the magnetic field is swept, the precession frequency of the magnetic system remains equal to the frequency of the exciting field. In this case, the signal amplitude increases significantly before the field reaches a critical value at which the signal is destroyed. A natural explanation for this effect was found in the works of Anderson and Suhl, in which the magnon system was presented as a nonlinear oscillator^31^. Recently this theory has found brilliant confirmation in experiments with a nanoscale YIG sample, which can really be represented as a single oscillator^32^. The properties of foldover magnetic resonance and its relaxation are also discussed in more detail in the article^33^. This article presents the experimental results of foldover resonance in a permalloy micro-strip. The article discusses in detail the analytical solution of the Landau–Lifshitz–Gilbert equations in conditions of nonlinear resonance in a micro-object.

However, great difficulties arose in attempts to quantitatively interpret the results of nonlinear magnetic resonance in macroscopic samples. The effective magnetic field in macroscopic samples is inhomogeneous due to edge effects, spatial inhomogeneity of the external field, and various types of local defects. Therefore, nonlinear magnetic resonance cannot be described in terms of a single resonator. Within the framework of the nonlinear resonance model, it must be described by a set of coupled oscillators. The theoretical analysis is complicated by the fact that resonance excitation is also spatially nonuniform, especially when it is excited by a strip line. In addition to the local Gilbert damping, one should also take into account the relaxation processes associated with the magnon density and spin diffusion, as well the interaction with local metallic strip line^33^. All these circumstances lead to the inadequacy of the experimental results of foldover resonance in macroscopic YIG films of the classical model, as shown in the detailed studies presented in^34^. To understand the contradiction between theory and experiment in this work, we conducted our own research in a more adequate way. The obtained experimental results show their excellent agreement with the mBEC theory and the presence of spin superfluidity.

## The experimenal results

We performed our investigations on X-band EPR spectrometers Varian E-12 and Bruker ELEXSYS E580 pulse spectrometer. The use of an EPR spectrometer instead of a strip line has several advantages. First, the excitation of resonance is spatially homogeneous. Secondly, the studies were carried out at a constant frequency and with a sweeping magnetic field. This eliminates the effects of changing RF line matching that affect the signal as the frequency changes. The resonator walls are located far from the sample. In the case of a strip line, heating of the sample may occur due to direct contact with the line and its overheating due to its miniature size. Finally, the electronics of the EPR spectrometer are highly sensitive. Therefore, the Q-factor of the resonator was damped to 80, which eliminated the influence of the resonator line on the magnetic resonance signals. As a result, our experiments excluded a number of factors that had an impact on the research results in^34^.

**Figure 1:**
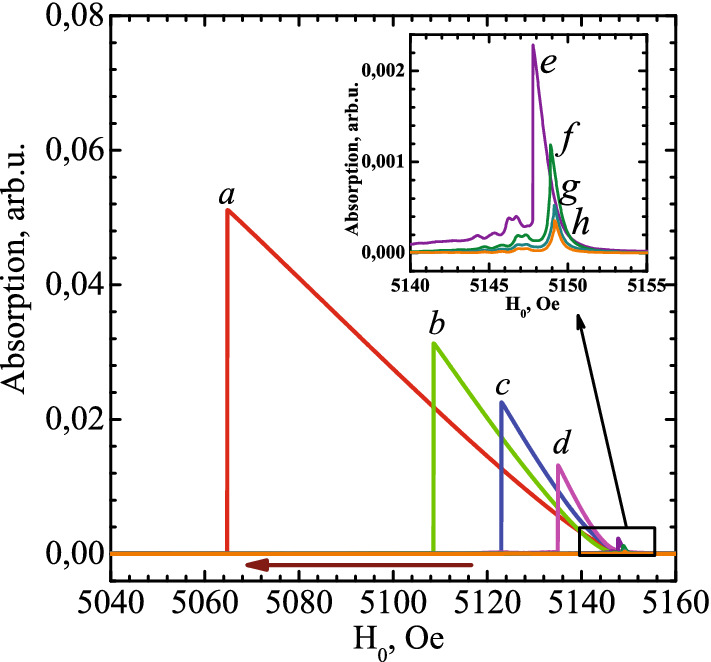
Amplitude of the absorption signal at different RF pump power in a decreasing magnetic field. The enlarged scale is shown in the inset. Here and in the next figures signals marked a–h corresponds to RF power of 80, 40, 20, 10, 1, 0.4, 0.1 and 0.05 mW, respectively for the disc of 0.5 mm.

**Figure 2:**
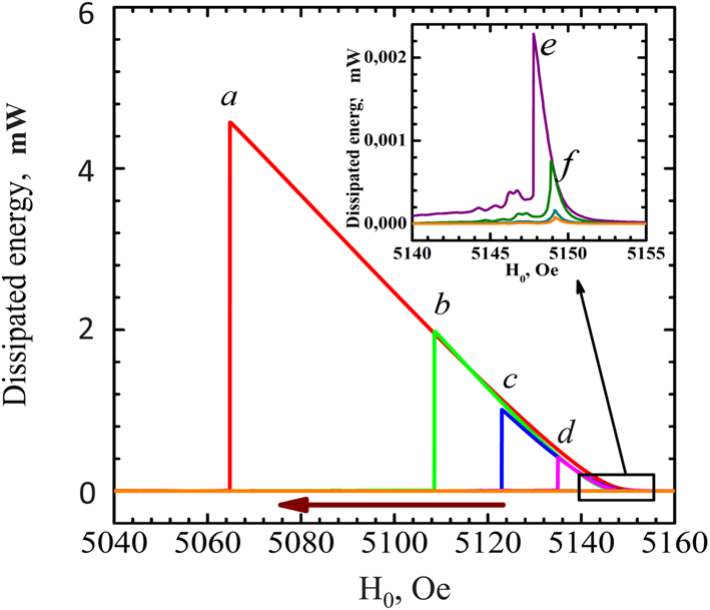
The energy dissipated by a magnon spin system at different level of exciting power. The energy was calculated as a product of absorption signal (Fig. 1) on the amplitude of RF field. The enlarged scale is shown in the inset.

The experiments were carried out on YIG films with a thickness of 6 $\mu$m in the form of discs with a diameter of 0.5 and 0.3 mm. Sample preparation is described in the Methods section. Signals from the first sample obtained on the Varian EPR spectrometer at a frequency of 9.26 GHz at room temperature and at different RF excitation powers are shown in Fig. 1. At first glance, they look like well-known nonlinear foldover resonance signals. Let’s analyze the power consumed by the magnetic system at different pump powers. To do this, we multiply the absorption signal by the root of the RF pump power. The result is shown in Fig. 2. It is clearly seen that the absorbed energy, like the mBEC state, does not depend on the pump power, but only on the displacement of the magnetic field from linear resonance (see Eq. 5). This is a distinctive property of the Bose condensate of magnons. This result for YIG films was obtained for the first time and is associated with the fact that the chemical potential of mBEC is determined by the frequency and not by the amplitude of excitation. On the contrary, in the classical theory of nonlinear oscillators, the state was determined by the amplitude of the excitation. In this graph, we are using a scale of dissipated energy, calibrated in watts. The calibration procedure will be explained below.

According to the theory of magnetic resonance in an out of plane magnetized YIG film, its frequency depends on the angle of magnetization deflection from the magnetic field $\beta$, which characterizes the density of non-equilibrium magnons^35^:

5 $$\begin{aligned} \omega _N = \omega _0 - \gamma 4\pi M{_S}\cos {\beta }, \end{aligned}$$

where $\omega _0 - \gamma 4\pi M{_S}$ is the frequency at low excitation, which is determined by the external field (the first term) and the demagnetizing field (second term). The frequency increases with the deviation of magnetization, that is, with an increase in the number of non-equilibrium magnons proportional to $(1-\cos {\beta })$. According to this formula, we can recalculate the observed shift of the magnetic field at which the nonlinear resonance takes place by the corresponding frequency shift which would be in a constant field. Moreover, the corresponding angle of magnetization deflection is easy to calculate from the frequency shift. Noteworthy is that at low excitation corresponding records f, g, h the absorption signal linearly depends on the amplitude of the RF field. Nonlinear shift of the field corresponding to the signal maximum appears starting from the record e and is about 2 Oe. In what follows we will refer to this phenomenon as the “field shift”. This shift corresponds to the angle of deflection of about 3$^\circ$. This angle corresponds to the condition for the onset of Bose condensation of magnons in the YIG film^23^. At high excitation, the quantum properties of mBEC should be taken into account.

In our experiments, we achieved a magnetic field shift of 80 Oe at an excitation power of 80 mW which corresponds to a frequency shift of about 220 MHz and an angle of magnetization deviation of about 18$^\circ$ as is found from Eq. (5)! Similar signals were observed on the second sample 0.3 mm in diameter, the amplitude of which was smaller in accordance with the sample size.

A distinctive feature of the foldover resonance is that its signal disappears at some critical field shift, which depends on the excitation power. In addition, a strong hysteresis of signal recovery is observed when scanning the field (frequency) in the opposite direction. Quantitative results of our experiment are completely understandable in a frame of mBEC formation, in contrast to the classical explanation^34^.

The reason for the disappearance of the signal is that the relaxation rate of magnons increases with an increase in their density and the corresponding angle of deviation of the magnetization. The signal disappears when the RF pump power becomes insufficient to maintain the required magnon density. For a signal to exist at large field shifts, an increase in the pump power is required. The energy, absorbed by the mBEC state, is proportional to $I_a H_{\mathrm{RF}}$, where $I_a$ is the signal of absorption and $H_{\mathrm{RF}}$ is the amplitude of RF field. In the precession frame, where both RF fields and deflected magnetization $M_\perp$ are constant, the interaction energy term is

6 $$\begin{aligned} F_{\mathrm{RF}}=- H_{\mathrm{RF}} M_{S \perp } \cos (\alpha -\alpha _{\mathrm{RF}}) ~, \end{aligned}$$

where $\alpha$ and $\alpha _{\mathrm{RF}}$ are the phases of magnetization precession and RF field. This term softly breaks the *U*(1)-symmetry and serves as a source of the mass of Nambu-Goldstone mode of mBEC^36^.

The power absorbed by mBEC from RF field pumping is:

7 $$\begin{aligned} W = \omega M_S H_{\mathrm{RF}}\sin \beta \sin {(\alpha -\alpha _{\mathrm{RF}})}. \end{aligned}$$

The mBEC precession phase can be self-adjusting so that the absorbed power compensates for the losses. The signal breaks down at the moment, when the RF power is not enough to compensate the magnons dissipation. Since the pumping (7) is proportional to $\sin \beta \sin (\alpha -\alpha _{\mathrm{RF}})$, a critical tilting angle $\beta _c$, at which the pumping cannot compensate the losses, increases with increasing $H_{RF}$. The breaks down should occurs when the phase shift ($\alpha -\alpha _{\mathrm{RF}}$) reaches 90$^\circ$. However, due to the inhomogeneity of relaxation, it can appear at a smaller average angle^24^.

**Figure 3:**
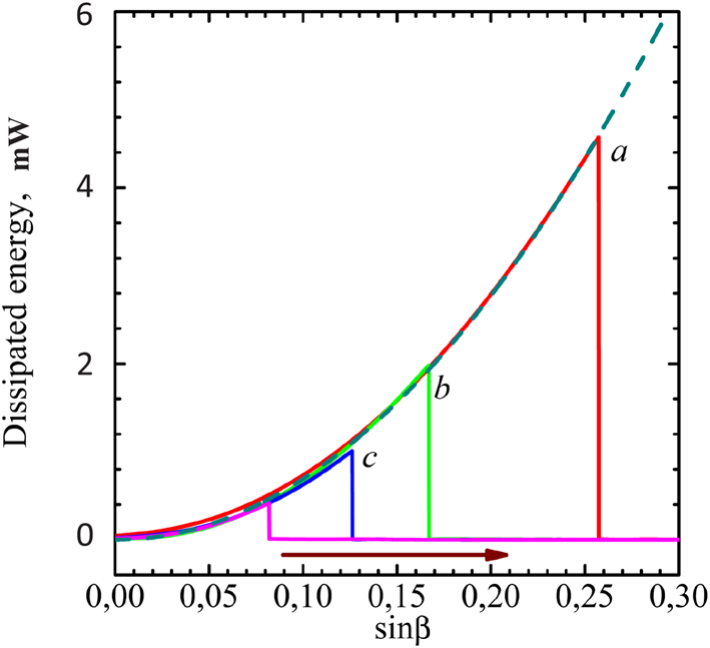
The dissipated energy as a function of angle of deflection for the 0.5 mm disc. The fitting lines correspond to square dependence.

The main process of magnetic relaxation in the YIG film is determined by Gilbert dumping, which is proportional to the deflection angle of the precessing magnetization $\beta$^37^. Usually the coefficient of Gilbert damping $\alpha _G$ is estimated from the homogeneous broadening of resonance line $\delta H/H$ at a small excitation, where $\delta H$ is the ferromagnetic resonance width, and H is the magnetic field at which the resonance is observed. . For our sample it is about $2 \times 10^{-4}$ as follows from the record *h* in Fig. 1. However, the method for estimating $\alpha _G$ from line broadening is not reliable, since in the case of macroscopic samples it can mix with inhomogeneous line broadening. From the result of our experiments we can suggest a new method of $\alpha _G$ estimation which is not sensitive to inhomogeneous broadening. The energy, dissipated by mBEC due to damping is equal to

8 $$\begin{aligned} W = \sigma \, \alpha _G^2 \,M_S^2 \, \sin ^2\beta ~ , \end{aligned}$$

where $\sigma$ is a spectrometer parameter that relates the pump power, the amplitude of the RF field, and the ratio of the cavity and sample volumes. The dissipated energy as a function of the magnetization deflection, recalculated from the field shift (Eq. (5)) is shown in Fig. 3. The perfect square dependence confirms that the main source of dissipation in our experiments is the Gilbert damping. The estimations of spectrometer parameter $\sigma$ correspond well to $\alpha _G$ of about $2 \times 10^{-4}$.

**Figure 4:**
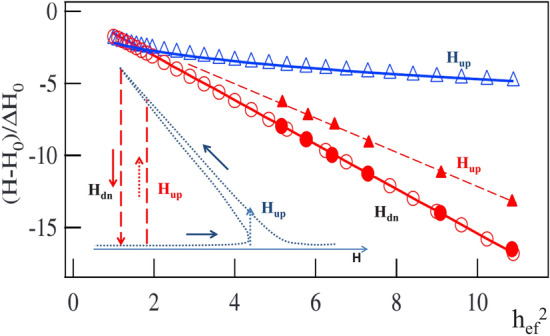
The dependence of fields H$_{dn}$ and H$_{up}$ in units of field shift divided by the line broadening ($\Delta H_0$) as it described in^33^ for a single oscillator and a microscopic sample (open symbols). We use this picture to show the principal difference with our results. The parameter $h_{ef}^2$ is used, which is proportional to the pumping power as a fitting for field H$_{dn}$ (filled symbols). It is clearly seen that the field H$_{up}$ has a much larger shift from the resonance field than what follows from the theory of a single nonlinear oscillator.

The foldover resonance is usually characterized by a big difference between the fields of signal dropout at a field sweep down ($H_{dn}$) and its recovering at a sweep field up ($H_{up}$)^33^. This follows from the property of a single non-linear oscillator, shown in inset in Fig. 4. The $H_{up}$ field corresponds to the end point of the second branch of the solution and is located relatively close to the linear resonance field. This model describes well the results of experiments with a nanosized YIG samples^32^. But this does not apply to macroscopic samples. In our case the difference between ($H_{dn}$) and ($H_{up}$) is much smaller as shown in Fig. 4 by the filled symbols. This difference can be explained by a non-resonance excitation, well known for mBEC in 3He-B^19^ and in solid-state antiferromagnets^29^. The similar effect was observed also in the in-plane magnetized YIG film^38^. It consists of the fact that the RF field excites spin oscillation modes with energies higher than homogeneous resonance. As a result, the magnon density increases and a magnonic BEC is formed. The distribution of mBEC from the excitation region to the lower magnetic field in a YIG film placed in a magnetic field gradient has recently been investigated and confirmed in work^39^.

### Conclusion

In this paper, we have shown that the problems of inadequacy between the theory of folded resonance and the experimental results demonstrated in^34^ are related to the fact that quantum effects must be taken into account. They emerge due to Bose condensation of non-equilibrium magnons at large angles of magnetization deflection. We carried out a series of experiments, in many respects similar to the previous one with $^3$He, in which Bose condensation of magnons and spin superfluidity were discovered. We have shown that an adequate theoretical description of the foldover resonance in macroscopic samples appears when the quantum properties of mBEC are taken into account.

Our model of mBEC formation is also confirmed by the results of pulsed experiments. Under pulsed excitation with a duration of less than 50 ns, the formation of mBEC was not observed. As we know from research in antiferromagnets^29^, the pulse duration must be sufficient to pump the required magnon density. The optimal pulse duration for the formation of mBEC in our experiments was 400 ns and more. In this case, we were able to detect a long-lived induction signal that unambiguously confirms the formation of a magnonic BEC^40^.

Measuring the Gilbert damping in macroscopic samples from the broadening of the resonance line is a serious problem, due to the influence of inhomogeneous broadening. Our article demonstrates a new method for measuring it. It is insensitive to inhomogeneous broadening of the resonance line due to the redistribution of magnons by spin supercurrent^25^. A quadratic dependence of the dissipation on the angle of deviation of the magnetization was demonstrated, consistent with theory. To use this method for the serial study of macroscopic samples, the parameters of spectrometer must be calibrated. In our case, the assessment of the spectrometer sensitivity showed good agreement with the Gilbert damping constant obtained from the resonance line at low power.

Our experimental results confirm a significant change in the properties of the folded magnetic resonance in YIG due to the quantum properties of mBEC and the formation of a state with coherent precession. This state can be thought of as a quantum object that can be used for quantum calculations. Entanglement between two BEC states was suggested by^41^, and the Josephson effect between two BECs was demonstrated in a superfluid $$ ^3$$ He-B recently^42^. The important point is that the mBEC qubit can be used even at room temperature. This state is the first superfluid state of condensed matter at room temperature. It is an ideal platform for development of microwave magnetic technologies, which have already resulted in the creation of the magnon transistor and the first magnon logic gate^43,44^. The YIG can be used as the basis for new solid-state quantum measurement and information processing technologies including cavity-based QED, optomagnonics, and optomechanics^45^. This discovery paves a way to many quantum applications, such as magnetic Josephson effect, long distance spin transport, Q-bit, quantum logics, magnetic sensors and others^26^. It can also be considered as a new branch of modern magnetism–supermagnonics.

### Methods

Samples of YIG films with a thickness of 6 $\mu$m were prepared by high-temperature liquid-phase epitaxy on GGG substrates with the (111) crystallographic orientation^46^. The substrates were 3 inches in diameter, 500 $\mu$m thick, and chemo-mechanically polished. To reduce the effect of cubic magnetic anisotropy, we used scandium substituted $Lu_{1.5}Y_{1.5}Fe_{4.4}Sc_{0.6}O_{12}$ iron garnet films; the introduction of lutetium ions was necessary to match the parameters of the substrate and film crystal gratings. It is known that the introduction of scandium ions in such an amount reduces the field of cubic anisotropy by more than an order of magnitude^47^. In addition, the used lutetium and scandium ions practically do not contribute to additional relaxation in the YIG. Next, a lithography procedure was carried out on the plates, followed by etching in orthophosphoric acid at a temperature of 180 C. Discs with a diameter of 500 and 300 $\mu$m were created, which were divided into separate samples using a scrambler. To avoid magnetic pinning on the surface the sample was etched in a hot phosphoric acid^48^. As a result, the edges of the disk have a slope of 45 degrees and had a smooth surface.
